# Dynamic, Morphotype-Specific *Candida albicans* β-Glucan Exposure during Infection and Drug Treatment

**DOI:** 10.1371/journal.ppat.1000227

**Published:** 2008-12-05

**Authors:** Robert T. Wheeler, Diana Kombe, Sudeep D. Agarwala, Gerald R. Fink

**Affiliations:** Whitehead Institute for Biomedical Research, Cambridge, Massachusetts, United States of America; Carnegie Mellon University, United States of America

## Abstract

*Candida albicans*, a clinically important dimorphic fungal pathogen that can evade immune attack by masking its cell wall β-glucan from immune recognition, mutes protective host responses mediated by the Dectin-1 β-glucan receptor on innate immune cells. Although the ability of *C. albicans* to switch between a yeast- or hyphal-form is a key virulence determinant, the role of each morphotype in β-glucan masking during infection and treatment has not been addressed. Here, we show that during infection of mice, the *C. albicans* β-glucan is masked initially but becomes exposed later in several organs. At all measured stages of infection, there is no difference in β-glucan exposure between yeast-form and hyphal cells. We have previously shown that sub-inhibitory doses of the anti-fungal drug caspofungin can expose β-glucan *in vitro*, suggesting that the drug may enhance immune activity during therapy. This report shows that caspofungin also mediates β-glucan unmasking *in vivo*. Surprisingly, caspofungin preferentially unmasks filamentous cells, as opposed to yeast form cells, both *in vivo* and *in vitro*. The fungicidal activity of caspofungin *in vitro* is also filament-biased, as corroborated using yeast-locked and hyphal-locked mutants. The uncloaking of filaments is not a general effect of anti-fungal drugs, as another anti-fungal agent does not have this effect. These results highlight the advantage of studying host–pathogen interaction *in vivo* and suggest new avenues for drug development.

## Author Summary

*Candida* is a common human commensal but disseminated candidiasis is a serious clinical problem, especially among immunocompromised patients. The innate immune system controls *Candida* infection, in part through the germline-encoded β-glucan receptor Dectin-1. However, during *in vitro* growth, *Candida albicans* mutes Dectin-1 recognition by cloaking its β-glucan underneath a layer of mannan. Bridging these two seemingly contradictory observations, we demonstrate that *C. albicans* masks β-glucan early during infection, but it becomes exposed later, allowing Dectin-1 to recognize the fungi and mediate immunity. Remarkably, treatment of mice with sub-therapeutic doses of the antifungal drug caspofungin causes exposure of β-glucan on *C. albicans* even when it would not be exposed naturally. We introduce a new technique for monitoring of epitope exposure during infection, which can be used to monitor the availability of any epitope for immune recognition. This technique allowed us to show that natural unmasking of β-glucan is not morphotype-specific, but drug-mediated unmasking is biased towards the invasive filamentous form of *C. albicans*. These results highlight the unexplored area of dynamic epitope exposure during infection and therapy, which might be targetable to enhance immune recognition and fungal clearance.

## Introduction

*Candida albicans*, a common commensal organism of humans, has emerged as an important fungal pathogen clinically due to the immunocompromised status of many patients as well as the ineffectiveness of current anti-fungal drugs [1]. The immune system has many pathways for recognizing and responding to *C. albicans*, including innate immune Toll-like receptors, lectin receptors, antibody, complement, and mannose binding lectin [2]. A major innate immune receptor for *C. albicans* is Dectin-1, a lectin that can recognize β-glucan, a unique component of the fungal cell wall. *In vitro* analysis shows that Dectin-1 recognizes fungal β-glucan (comprising glucan polymers with mixed β1,3- and β1,6- linkages), and signals through unique pathways to induce phagocytosis, up-regulation of immune killing mechanisms, and production of pro-inflammatory cytokines [3].

The mechanism of β-glucan signaling through Dectin-1 is unclear because this signature molecule on *Candida* and other fungi is enveloped by a cell wall mannoprotein layer that masks almost all of the β-glucan from immune recognition and mutes the host immune response [4],[5],[6],[7],[8]. On one hand, the β-glucan masking observed *in vitro* runs contrary to an active role for Dectin-1 recognition of β-glucan during infection [8],[9],[10]. On the other hand, *in vitro* Dectin-1 is clearly able to mediate protective responses to fungi and zymosan, a treated fungal particle with exposed β-glucan [9],[11],[12],[13],[14]. In addition, work in knock-out mice suggests at least a conditional requirement for Dectin-1 in resistance to the SC5314 strain of *C. albicans*, which may be dependent on mouse strain and/or *C. albicans* strain [13],[14].

An additional complexity at the *Candida*-host interface is that *C. albicans* has several developmental cell types including yeast, pseudohyphae, and hyphae, each with different modes of interaction with innate immune cells [15],[16]. The ability of *C. albicans* to switch between the yeast and filamentous forms is strongly associated with virulence. Filaments are distinct from yeast-form cells in cell wall structure, cell wall proteins and transcriptional programs. In addition, the hyphal form of the fungus has been shown to cause more tissue damage than the yeast-form fungus in *ex vivo* models of candidiasis [17],[18],[19]. Furthermore, immune recognition of yeast provokes a different immune response compared to recognition of hyphae [15],[20]. Intriguing recent work has suggested that only yeast-form cells possess exposed β-glucan at a few sites [9], but this has yet to be shown *in vivo*.

Although β-glucan can be an important signal for innate immune cells *in vitro*, and the β-glucan receptor Dectin-1 protects against fungal disease in mice, we still understand little of the dynamics of β-glucan-Dectin-1 interaction during infection or drug treatment. We have developed a new technique to directly measure morphotype-specific β-glucan exposure in mouse tissue during infection and anti-fungal treatment. We find unexpected dynamics of β-glucan exposure during infection and during treatment with the cell wall-directed drug caspofungin. Caspofungin treatment, either *in vitro* or *in vivo*, causes dramatic, hyphal-biased exposure of β-glucan. By contrast, although in the absence of the drug there is progressive unmasking of β-glucan during infection, there is no difference between yeast form and hyphal cells. Our results support a role for Dectin-1 in recognition of *C. albicans* during infection and drug therapy and demonstrate a novel morphotype bias in the action of the anti-fungal caspofungin, highlighting new avenues for drug development.

## Results

### Drug-induced β-glucan unmasking and fungicidal activities are filament biased

**Figure 1:**
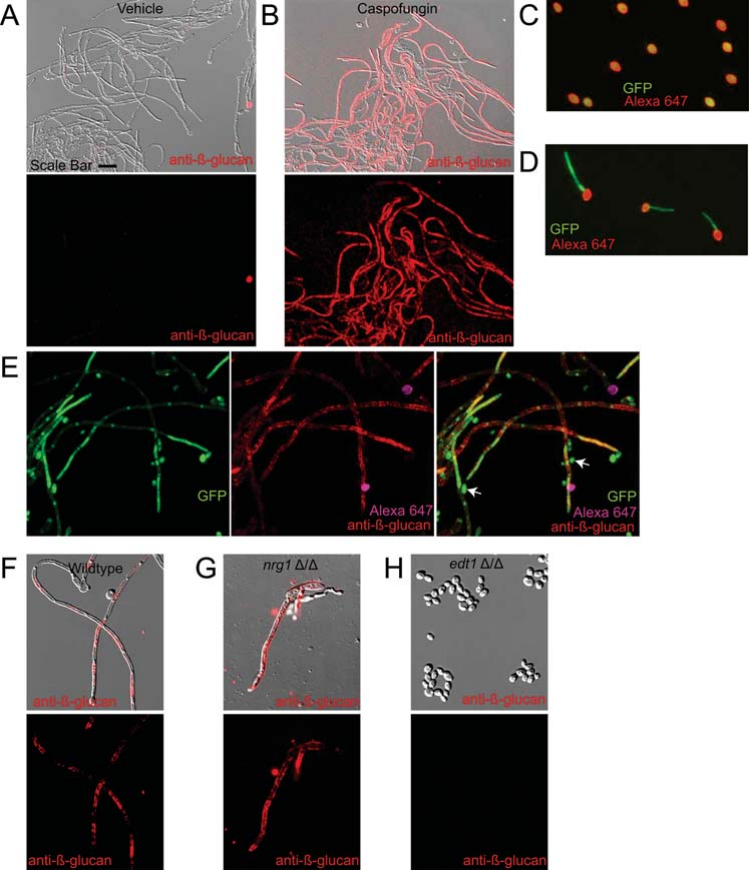
Caspofungin-mediated unmasking *in vitro* is filament-biased. (A,B) Wildtype strain CAF2-1 was grown overnight in RPMI-PS with vehicle (A) or sub-inhibitory doses of CF (B), then stained with anti-β-glucan antibody. Overlays between DIC and anti-β-glucan staining (in red) show that there is no β-glucan exposure on yeast-form cells, but there are low to high levels of exposure on filaments. (C) Covalent labeling of live yeast-form cells with Alexafluor 647-succinimidyl ester is homogeneous and bright. (D) Extensive growth *in vitro* of Alexafluor 647-labeled yeast-form cells in the presence of serum demonstrates that the label is long-lasting and remains confined to initial yeast-form cell wall. (E) Wildtype strain SC5314-GFP was pre-labeled with Alexa647, then grown overnight in RPMI-PS with subinhibitory doses of CF (½ MIC_50_). (F–H) Wildtype (F) or *nrg1*Δ/Δ (G) or *edt1*Δ/Δ (H) mutant cells pre-grown in YPD were washed and diluted into RPMI-PS plus different concentrations of CF; cells grown in ½ MIC_50_ of CF were stained with anti-β-glucan antibody and Cy3-labeled secondary antibody. Images are representative of three independent experiments. Scale bar shown in panel (A) is 20 microns long for panels (A) and (B) and 10 microns long for (C–H).

The anti-fungal drug caspofungin (CF) unmasks β-glucan at sub-inhibitory concentrations during growth of *Candida albicans* in culture media that promotes either exclusively yeast-form or hyphal-form growth [8]. During infection, *C. albicans* grows in conditions that promote both yeast and hyphal forms [21]. To study conditions more similar to *in vivo* growth, we grew *C. albicans* in sub-inhibitory concentrations of CF in RPMI media at a temperature that concurrently permit yeast-, pseudohyphal-, and hyphal-form growth. Growth at ½ the minimum inhibitory concentration (MIC) of CF causes striking β-glucan unmasking, as assayed with anti-β-glucan antibody specific for β1,3-glucan (Figure 1A and 1B).

Consistent with our previous work [8], there is very little cell death during growth in ½ MIC of CF, as assayed by propidium iodide staining. This result was further confirmed by using the WT-GFP strain, engineered to constitutively express yEGFP in the SC5314 clinical isolate. WT-GFP fluoresces at similar intensity to CAI4-GFP [22] and grows at wildtype rates (data not shown). The cytoplasmic GFP fluorescence enables the identification of live cells, because live cells have a characteristic cytoplasmic pattern of green fluorescence (see Methods).

Unexpectedly, there is a striking bias in exposure of filaments as opposed to yeast-form cells—a high percentage of hyphal cells have exposed β-glucan, whereas only a few yeast-form cells have exposed β-glucan (Figure 1E). The filament-specific exposure of β-glucan was also found in several clinical isolates, suggesting this is a general feature of caspofungin (Figure S1). To ensure that this differential effect on filaments was not due to persistence of the initial inoculum of yeast-form cells (which were pre-grown in the absence of CF), we followed the persistence of these original cells during CF treatment by marking them with the amine-reactive dye Alexa fluor 647-succinimidyl ester. As shown in Figure 1C, treatment of live cells with the dye homogeneously labels all cells in the inoculum. The label is retained on the surface of the original dye-tagged cells even when they are grown in hyphal-promoting conditions (37°C with serum), which results in no loss of label intensity on the mother cell and no transferral of label to growing hyphae in those cells (Figure 1D). This suggests that the cell surface proteins labeled by the amine-reactive Alexa fluor 647 dye are tightly retained in the cell wall and provide a long-lasting, non-dispersing mark for following the ancestry of C. albicans cells. We then used this to ask whether the yeast cells with no exposure are from the pre-culture or grew in the presence of caspofungin. As shown in Figure 1E, the majority of yeast-form cells in overnight cultures have no Alexa label and therefore were newly formed in the presence of CF. Thus, growth in pseudohyphal or hyphal form, as compared to yeast-form, predisposes *C. albicans* to β-glucan unmasking by CF.

The above data suggest that CF specifically unmasks hyphal forms. To further test this we utilized *C. albicans* mutants that grow exclusively in hyphal- or yeast-form, independent of growth media. Due to the possibility that any given mutant might show altered sensitivity unrelated to its morphotypic defect, we chose a variety of strains. Specifically, we analyzed two mutants locked in filamentous growth form (*tup1*Δ/Δ [23] and *nrg1*Δ/Δ [24]), two mutants locked in yeast-form growth (*edt1*Δ/Δ and *efg1*Δ/Δ *cph1*Δ/Δ [25]), and several wildtype strains (SC5314, CAF2, and WT-GFP). The *EDT1* gene, originally isolated based on its requirement for filamentation, results in constitutive yeast-form growth when deleted (Chen et al, unpubl. results). When these morphotype specific mutants grown at sub-inhibitory doses of CF are stained for β-glucan exposure, filament-locked mutants such as *nrg1*Δ/Δ have exposure similar to wildtype filamentous cells but yeast-locked cells such as *edt1*Δ/Δ show little exposure (Figure 1F–1H). The finding that yeast-locked cells show no β-glucan unmasking at sub-inhibitory CF concentrations supports the conclusion that morphogenesis and sensitivity to unmasking are linked.

**Figure 2:**
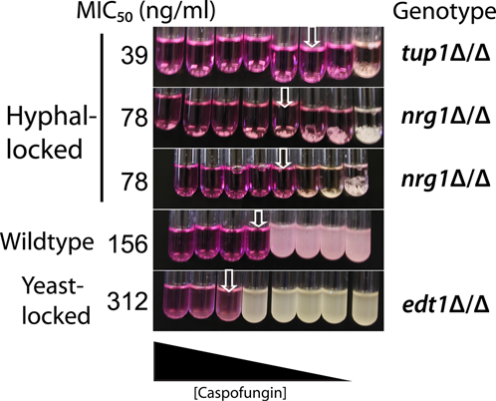
Caspofungin is more effective against filament-locked mutants than against yeast-locked mutants. Strains were pre-grown in YPD, washed, sonicated, diluted into RPMI-PS containing two-fold dilutions of CF, and grown overnight at 30°C to promote both yeast- and filamentous-form growth. RPMI has phenol red, so yellow color in the media indicates metabolic activity and acidification of the media. Changes in color and visual scoring of growth were used to identify MICs for each strain. Results are representative of four independent experiments run in duplicate.

The filament bias of β-glucan exposure by CF raised the possibility that the drug's fungicidal activity may also act preferentially on filamentous cells. To test this, we probed the efficacy of CF against *Candida* mutants locked in either the yeast or filamentous form under conditions that normally permit growth of both filaments and yeast-form cells. Compared to wild type strains, mutant filamentous strains consistently show greater drug sensitivity and yeast-form mutants show greater resistance (Figure 2). Because filamentation confers greater sensitivity to the fungicidal activity of CF, this suggests that both the β-glucan unmasking activity and fungicidal activity of CF preferentially affect filamentous cells *in vitro*.

### Direct measurement of baseline β-glucan exposure during infection with *ex vivo* immunofluorescence

Our *in vitro* results point to hyphal-biased activity of CF, but their relevance for understanding the drug's activity during therapy was unclear. We therefore developed a method that permitted us to isolate and assay the surfaces of live fungi for β-glucan exposure directly from infected tissue without prior fixation or sectioning. The fixation and sectioning procedures normally used to assay cell surface molecules could be misleading as fixation can itself alter cell surface structure and exposure while sectioning artificially exposes underlying molecules. In this technique, called *ex vivo* fluorescence (EVF), tissue homogenates obtained from infected animals are washed and whole cells are stained directly without fixation in order to preserve the live cell wall organization. Use of WT-GFP *C. albicans* permitted both the unambiguous detection of fungi amidst non-fluorescent kidney tissue and the identification of live fungi based on characteristic fluorescence pattern (see Methods).

**Figure 3:**
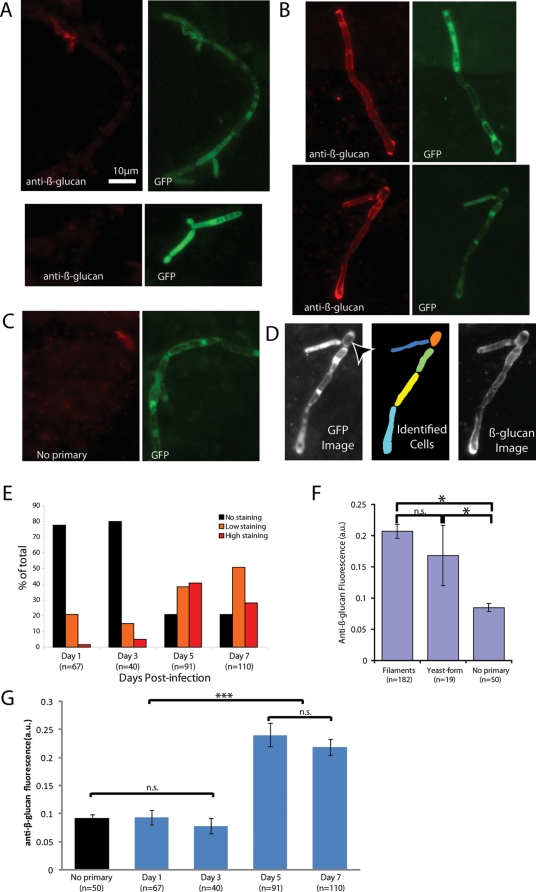
*Candida* β-glucan is masked early but unmasked late during disseminated infection. BALB/c mice were infected i.v. tail vein with 1×10^6^ (Day 1), 2.5×10^5^ (Day 3), 1×10^5^ (Day 5 or 7) organisms of strain SC5314-GFP. Animals were euthanized 1, 3, 5, or 7 days post-infection, kidneys were isolated, and organisms were stained by EVF for anti-β-glucan with or without primary antibody. (A) Anti-β-glucan staining of hyphae and yeast-form cells at Day 1 post-infection. Two representative examples are shown. (B) Anti-β-glucan staining of hyphae and yeast-form cells at Day 5 post-infection. Two representative examples are shown. (C) Representative filament from Day 5 post-infection, that the primary antibody was omitted. (D) Example of cell segmentation and quantification, with arrowhead pointing to yeast-form cell: Left panel is GFP image; center panel shows different cell segments in different colors; right panel shows the unmodified anti-β-glucan image used for quantification. (E) Categorization of exposure at different time points post-infection based on quantitative staining measure. No Staining is defined as within 2 standard deviations of the average fluorescence/cell of cells stained with no primary antibody; Low Staining is defined as cells with average fluorescence/cell greater than 2 standard deviations above, but less than 2-fold above, the average fluorescence of the no primary control; High Staining are cells with fluorescence/cell greater than two-fold higher than the average of the no primary control. These preparations have a significant amount of highly fluorescent debris from kidney tissue, which is the likely source of many of the 20% of cells that have “greater than background” levels of staining at the early time points. (F) Level of β-glucan exposure in hyphal cell wall vs. yeast-form cell wall at 5 to 7 days post-infection. Histogram shows the average fluorescence/cell for all cells quantified at 5 and 7 days post infection, in comparison to cells stained with no primary anti-β-glucan antibody, as measured in arbitrary units (a.u.). Error bars show standard error of average fluorescence/cell. All pair-wise comparisons were analyzed by two-tailed Student's T-test in Excel (Microsoft Corp.); asterisks indicate statistically significant differences (p<0.01). (G) Overall quantification of β-glucan exposure in all cells at Days 1, 3, 5, and 7 post-infection, as compared to background staining without primary antibody. All cells from a given day were pooled, averages and standard error for each group was calculated, and pairwise comparisons were made by two-tailed Student's T-test in Excel (Microsoft). Day 1 and Day 3 groups show exposure indistinguishable from the No Primary group (p>0.35 for all pairwise comparisons), and Day 5 and Day 7 groups are indistinguishable from each other (p>0.35), but all comparisons between the two groups show significant differences (p<0.000001). Comparisons where differences are not significant (p>0.35) are indicated with n.s. and significant comparisons (p<0.000001) are indicated with three asterisks. Data shown in panels A–G are derived from 12 experiments (Day 1) or 2 experiments each (Days 3, 5, and 7) which were internally consistent. Scale bar in (A) is 10 microns long and applies to all images.

To provide a baseline against which to assay the affects of CF, the course of β-glucan exposure during disseminated infection in the absence of CF was measured in BALB/c mice infected intravenously with WT-GFP *Candida*. Fungal cells were subsequently identified in their kidney homogenates stained by EVF using a commercially available antibody directed against β-glucan. As shown in Figure 3A, EVF-stained *C. albicans* isolated from the kidney within 16 hours post-infection did not have appreciable β-glucan exposure (Figure 3A). Similar data were found in heart and brain homogenates (data not shown). The masking of β-glucan on *Candida* at early times post-infection in all cell types suggests that Dectin-1 plays a minimal role in recognition at this time during infection.

Dectin-1 is reported to have a role in clearance of fungi late in infection [14], so β-glucan recognition by this receptor might not normally play a role until later during infection. To discover whether β-glucan is unmasked later during infection, we infected mice intravenously with lower doses of WT-GFP *C. albicans*, euthanized animals at 1, 3, 5, and 7 days post-infection, and examined β-glucan exposure by EVF. Remarkably, by five days post-infection a large percentage of cells had exposed β-glucan (Figure 3B). The anti-β-glucan antibody is a mouse monoclonal IgG antibody; however, the control without primary antibody (Figure 3C) was negative, showing that no detectable host IgG was bound to the fungus during growth in kidney tissue. In addition to this control, the robustness of our staining protocol was tested using different incubation times, omitting the blocking reagent (bovine serum albumin), and testing two different isotype control antibodies to ensure this assay does not introduce artifactual staining (Figures S2, S3, and S4). To quantify the progression in β-glucan exposure, cells were identified and cell wall-associated β-glucan fluorescence was quantified as shown in Figure 3D using the CellProfiler software package [26]. As shown in Figure 3E, the percentage of cells with β-glucan exposure increases from approximately 20% to 80% over the time course, demonstrating time-dependent unmasking of β-glucan during infection.

To assess the cell-type specificity of natural β-glucan unmasking *in vivo*, we compared exposure between the yeast-form and hyphal-form cell wall at early and late points during infection. As shown in Figure 3F, there was no overall difference in unmasking between cell forms. Overall, there is very little β-glucan available for immune recognition in either cell type early during infection but there is significant exposure of both cell types as infection progresses. These data demonstrate that, during infection, *C. albicans* β-glucan is initially masked from immune recognition, but, over time, becomes unmasked in a morphotype-independent fashion.

### Caspofungin treatment during *C. albicans* systemic infection causes β-glucan unmasking

**Figure 4:**
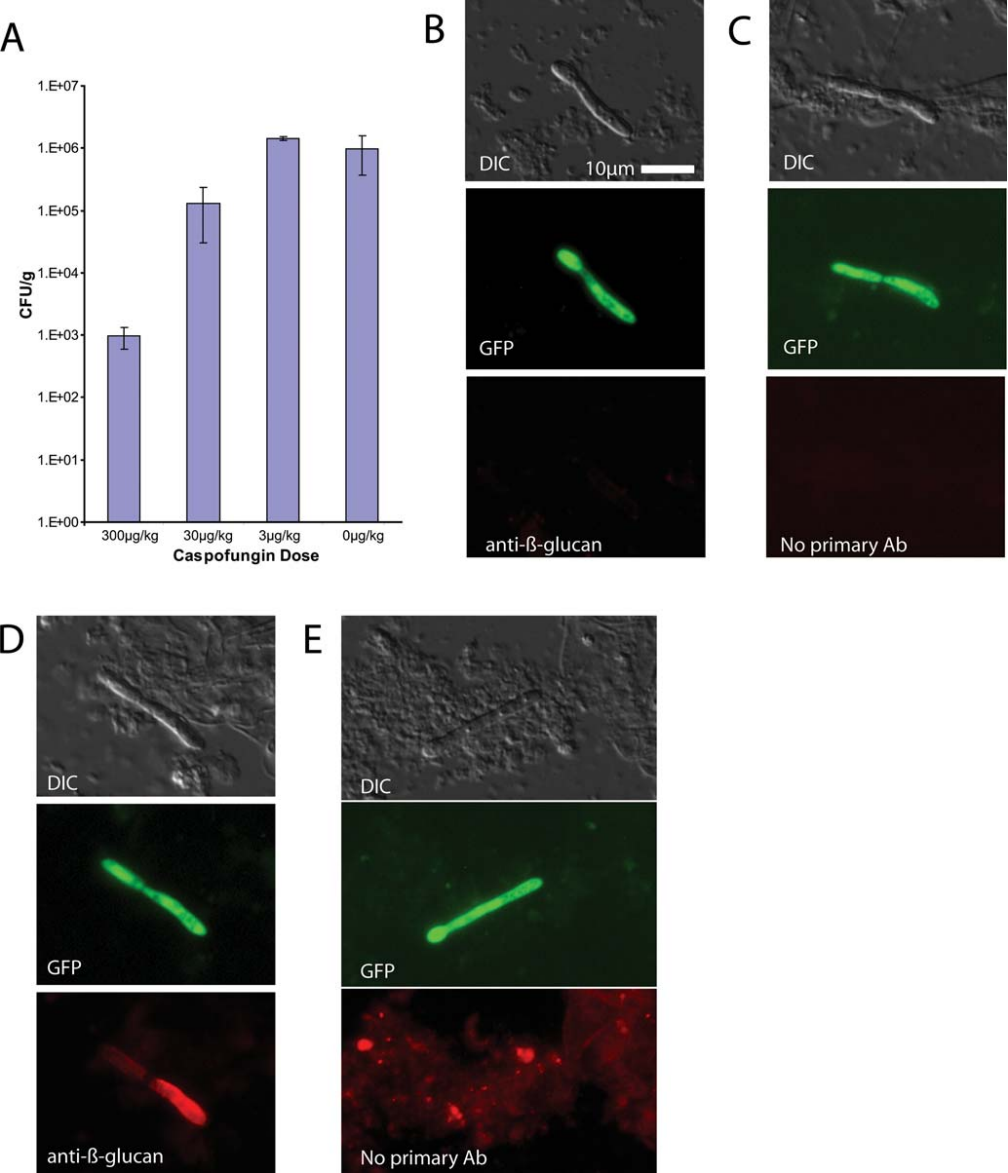
Treatment of *C. albicans* with caspofungin unmasks β-glucan. BALB/c mice were injected i.p. with caspofungin (CF) or vehicle and then immediately infected i.v. tail vein with strain SC5314-GFP. 16 hours post-infection, mice were euthanized and kidneys were dissected then homogenized. (A) Kidney homogenates were assayed for viable colony forming units by broth dilution and plating to YPD agar plates. Bars represent the average of triplicate plates, and error bars represent standard deviations. (B–E) Kidney homogenates were stained with or without anti-β-glucan monoclonal antibody and then with Cy3-labeled secondary antibody as described in Materials and Methods. (B,C) SC5314-GFP isolated from mice treated with vehicle. (D,E) SC5314-GFP isolated from mice treated with 30 µg/kg CF. Images and CFU data are representative of more than 12 experiments using between one and four mice per treatment group. Scale bar in (B) is 10 microns long and applies to all images.

To determine if treatment of disseminated candidiasis with caspofungin (CF) could artificially increase β-glucan exposure, we used different levels of CF to identify a sub-therapeutic dose (30 µg/kg) that would allow isolation of live fungi that had grown in the presence of drug (Figure 4A). These concentrations are in line with those observed to be sub-inhibitory by other investigators (C. Douglas, pers. comm.) We then infected mice with WT-GFP Candida, isolated kidneys at 16 hr post-infection when all β-glucan should be masked, and stained fungi with EVF to look at β-glucan exposure. Fungi isolated from mice treated with intermediate doses of CF had very high levels of β-glucan exposure (Figure 4D), in contrast to *C. albicans* isolated from mice treated with vehicle (Figure 4B). The no-primary-antibody controls (Figure 4C and 4E) show that this increase is not due to bound host IgG.

**Figure 5:**
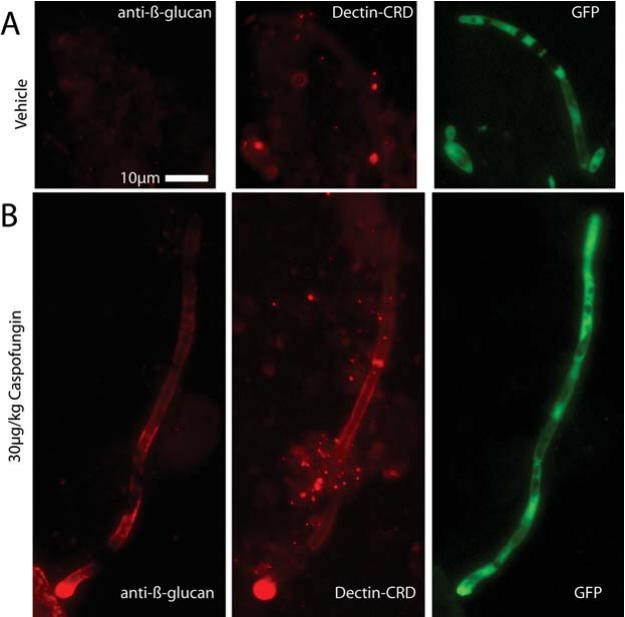
Unmasking by caspofungin exposes epitopes recognized by the Dectin-1 β-glucan receptor. Mice were infected and treated as in Figure 4. At 16 hr post-infection, kidney homogenates were stained with anti-β-glucan antibody followed by Cy3-labeled secondary antibody and Alexafluor 647-labeled recombinant Dectin-1-CRD. (A) *Candida* isolated from vehicle-treated mouse (B) *Candida* isolated from mouse pre-treated with 30 µg/kg CF. Images are representative of more than 6 experiments using between 1 and 3 mice per treatment. Scale bar in (A) is 10 microns long and applies to all images.

To test if this increase in β-glucan exposure has biological relevance for immune recognition, we probed with the purified carbohydrate recognition domain of the β-glucan receptor Dectin-1 (Dectin1-CRD). As shown in Figure 5, the increased β-glucan exposure is accompanied by increased availability of β-glucan for immune recognition (Figure 5). In each of the other types of experiments presented, Dectin-CRD binding was assayed and found to mirror antibody binding (data not shown). Thus, treatment with CF results in exposure of the pro-inflammatory β-glucan epitope and increased binding to the innate immune receptor Dectin-1.

**Figure 6:**
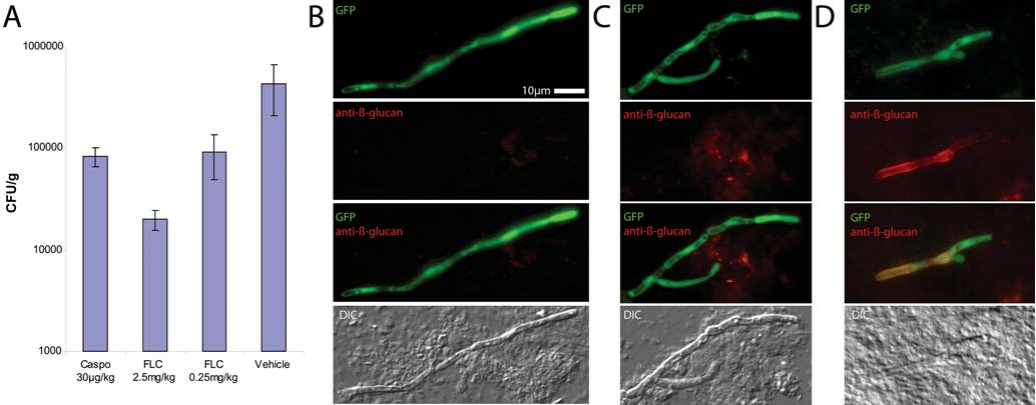
Fluconazole treatment does not unmask β-glucan. Mice were pre-injected with fluconazole and infected as in Figure 4. At 16 hr post-infection, kidneys were homogenized. (A) Homogenates were diluted and assayed for viable colony forming units or (B,D) homogenates were stained with anti-β-glucan monoclonal antibody and Cy3-labeled secondary antibody. Treatments of mice before infection were the following: (B) single dose of PBS (vehicle), (C) single dose of 2.5 mg/kg fluconazole in PBS, (D) single dose of 30 µg/kg caspofungin in PBS. Images and CFU data are representative of two independent experiments with 2 to 3 mice per group. Bars in (A) represent averages of triplicate counts and error bars represent standard deviations. Scale bar in (B) is 10 microns long and applies to all images.

To determine whether β-glucan unmasking is a consequence of treatment with another known anti-fungal compound, we treated WT-GFP-infected mice with sub-therapeutic doses of either fluconazole, an anti-fungal drug that targets sterol biosynthesis, or CF, and then examined β-glucan exposure 16 hours post-infection (Figure 6). In this experiment, both drug treatments are active and result in 10-fold decreased fungal burden (Figure 6A) but only CF treatment causes β-glucan exposure (Figure 6B–6D). Thus, β-glucan unmasking is specifically associated with CF treatment and is not a default stage in killing of *C. albicans*.

### β-glucan exposure *in vivo* preferentially affects the filamentous cells

**Figure 7:**
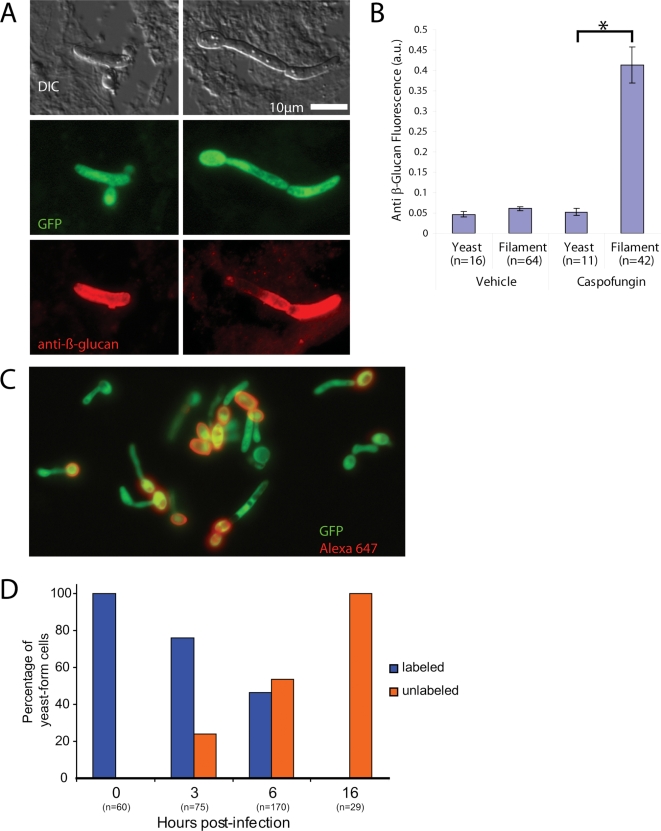
Caspofungin-mediated unmasking *in vivo* is filament-biased. (A) Two examples in which yeast-form cells have very little exposed β-glucan, in contrast to the filamentous cell bodies. SC5314-GFP was isolated 16 hr post-infection and stained as above. (B) Quantification of exposure of hyphae and yeast-form cells in vehicle and CF-treated cases. Images are representative of more than 12 experiments. Quantification includes data from two independent experiments and includes 133 individual cell segments. Error bars represent standard deviations. All pair-wise comparisons were analyzed by two-tailed Student's T-test (Excel, Microsoft Corp.); asterisks indicate statistically significant differences (p≤0.0001). (C) Growth pattern *in vivo* is similar to *in vitro*, and proliferation leads to some unlabeled yeast-form cells already by 3 hours post-infection. (D) Quantification of labeling over time shows that, by 16 hr post-infection, no labeled cells are visible. Quantification includes data from two or three independent experiments per time point, although 100% labeling efficiency was always confirmed before infection. Scale bar in (A) is 10 microns long and applies to all images.

The potent β-glucan unmasking activity of caspofungin *in vivo* provided a foundation for looking at the morphotype dependence of β-glucan unmasking during treatment of disseminated infection. Examination of individual cells for β-glucan exposure due to CF showed that filament cell walls are exposed, but yeast cell walls are not (Figure 7A). Quantification with CellProfiler demonstrates the robustness of this phenomenon, in which filamentous cells stain with anti-β-glucan whereas yeast-form cells show undetectable levels of β-glucan exposure (Figure 7B). For *Candida* isolated from vehicle-treated mice, neither morphotype showed detectable β-glucan exposure at these times after infection. Dectin-CRD binding closely paralleled anti-β-glucan staining for both caspofungin and vehicle treatment (data not shown).

To ensure that this differential effect on filaments was not due to persistence of the initial inoculum of yeast-form cells, we Alexa fluor-labeled fungi pre-infection and followed the appearance of unlabeled yeast-form cells (Figure 7C and 7D). As shown above in Figure 1, this covalent labeling technique tags cell surfaces with a long-lasting, non-dispersing mark for following the ancestry of cells. By three hours post-infection, there was already significant appearance of unlabeled yeast-form cells, which grew exclusively post-labeling and therefore post-infection. By six hours, there were approximately equal numbers of labeled and unlabeled cells, and by 16 hours there were no labeled cells. Notably, at the six hour time point, the fluorescence of labeled cells was undiminished, whereas the percentage of labeled cells declined, confirming *in vitro* experiments showing that the label is very stable. This quantitatively demonstrates that the cells from the initial inoculum are all cleared by 16 hours post-infection. Therefore, yeast- and hyphal-form cells examined at 16 hours post-infection all grew in the presence of the same level of CF and the filament bias in β-glucan exposure results from the differential action of the drug on different cell types.

## Discussion

A critical goal in understanding both host-pathogen interaction and drug action is to probe their dynamics during infection. *In vivo* experiments show that cell wall β-glucan is a key fungal signature molecule used by the innate immune system to clear fungal infection, yet *in vitro* experiments suggest that *Candida albicans* masks β-glucan from immune recognition. To resolve this apparent paradox, we developed and exploited an *ex vivo* fluorescence (EVF) technology to measure the changes in exposure of β-glucan in live cells during infection. This procedure showed that in the normal course of infection β-glucan is initially masked but subsequently exposed on the surface of *C. albicans*. This reconciles *in vitro* data that *C. albicans* β-glucan is masked by mannoprotein [8],[9] with *in vivo* infection data suggesting a potential role for Dectin-1 in immunity to *C. albicans*. *In vivo* data with knockout mice is inconsistent– clearance of the SC5314 strain of *C. albicans*, used in this study, from mice in the mixed BL/6×129/Sv background, is dependent on Dectin-1 [14], whereas clearance of a different strain of *C. albicans* from mice in the BL/6 background does not require Dectin-1 for efficient clearance [13]. Taken together, these data suggest that the Dectin-1 β-glucan receptor plays a role in resistance to *C. albicans* in conditions most similar to those used in our study [14]. Although it is not known how this unmasking occurs during the course of the infection, it is possible that, with time, immune cells accumulate in sufficient numbers to directly damage the cell wall and expose β-glucan. Infiltrating immune cells may also create an environment that induces physiological defenses in the fungus, resulting in altered cell wall signaling and increased exposure of β-glucan. Innate immune cells with the capacity to damage *Candida* are recruited to the site of infection in the first several days of infection, favoring these models [21],[27],[28]. Alternatively, there may be a novel pathogen-intrinsic pathway late in infection which is programmed to alter architectural changes independent of immune function.

EVF also permits unambiguous identification of cellular morphotype, allowing a rigorous analysis of β-glucan exposure in yeast vs. hyphal cells. In the absence of drug there is no difference in exposure of β-glucan between yeast-form and hyphal cells: both are effectively masked from the immune system early during infection and both are unmasked later. This provides evidence that during systemic infection of tissues, filamentous growth does not allow the fungus to evade recognition by the Dectin-1 β-glucan receptor. Previous work with *in vitro*-grown *C. albicans* using different media to promote morphotype-specific growth had suggested that hyphal cells are uniquely immune from Dectin-1-mediated recognition [9]. However, our *in vivo* results show clearly that during infection there is no morphotype-specific masking or unmasking of β-glucan. This contrast highlights the critical advantage of looking at the host-pathogen interaction during infection rather than *in vitro*.

Treatment of mice with CF during disseminated infection unmasks *C. albicans* β-glucan and makes it available for immune recognition by the Dectin-1 β-glucan receptor. Although unmasking during the normal course of infection shows no morphotype specificity, CF-mediated unmasking is filament-biased and both β-glucan unmasking and fungicidal activities of CF are directed more at filaments than yeast-form cells. The bias may be due to intrinsic compositional differences between hyphal and yeast wall [29],[30],[31],[32] or due to differences in transcription of cell wall regulators sensitive to caspofungin [33]. Filament bias may contribute to the clinical efficacy of CF by selecting for yeast-form growth which is less invasive, more easily cleared and stimulates a more protective immune response [15],[20]. The finding that a particularly effective anti-fungal drug has morphotype bias opens the possibility of new anti-fungals designed for increased effectiveness against the filamentous, invasive form of *C. albicans*. The morphotype bias also highlights the potential shortcomings of using a single growth condition to determine *in vitro* drug effectiveness, which can favor one morphotype over another and fail to reveal such biases.

Our findings draw attention to a relatively unexplored avenue for drug development—the identification of drugs that do not affect the growth or viability of the pathogen *per se* but render the pathogen more susceptible to the host immune system. It is likely that clinical use of CF also causes increased recognition through the β-glucan receptor, making hyphal cells more susceptible to immune attack and contributing to the success of the drug. Recent work shows that caspofungin causes unmasking in other fungal species and makes them more susceptible to immune attack, underlining the broad-spectrum robustness of this drug's activity to expose β-glucan even in the absence of classical fungicidal activity [4],[6]. Development of new anti-fungal drugs that specifically cause β-glucan unmasking may reveal novel classes of drugs that target a non-essential process, provide broad-spectrum clinical protection, and do not select for resistance in the absence of immune pressure.

## Materials and Methods

### *C. albicans* strains and growth conditions

*C. albicans* strain information is summarized in Table 1. *C. albicans* was routinely grown at 37°C in YPD media (DIFCO; 20 g/L peptone, 10 g/L yeast extract) containing 2% glucose. For infections, *C. albicans* was washed, counted by hemacytometer, and diluted to 1*10^7^/ml. For MIC experiments, *C. albicans* was pre-grown in YPD at 30°C overnight, washed, sonicated briefly with a Branson sonicator (3×10 seconds at setting of 1.5), and diluted 1∶100 to a concentration of 2.5*10^6^/ml (for WT and yeast-locked strains) or 1∶20 (for hyphal-locked strains that grow poorly) into RPMI-PS, RPMI (Invitrogen) containing phenol red, sodium pyruvate, HEPES pH 7.2, with added penicillin-streptomycin. Caspofungin (Cancidas intravenous formulation, Merck & Co., Inc.) was made fresh from lyophilized stock and diluted into RPMI-PS for *in vitro* studies or into PBS for intraperitoneal (i.p.) injection into mice (see below).

**Table 1:** Strain details.

Strain Name	Reference	Genotype
SC5314	[35]	Wildtype
CAN89	This paper	Wildtype, clinical isolate
CAN91	This paper	Wildtype, clinical isolate
CAN92	This paper	Wildtype, clinical isolate
CAF2-1	[35]	*ura3*Δ*::imm434/URA3*
*nrg1*Δ/Δ (MMC3)	[24]	*ura3*Δ*::imm434/ura3*Δ*::imm434 nrg1*Δ*::hisG nrg1*Δ*::hisG::URA3::hisG*
*efg1*Δ/Δ *cph1*Δ/Δ (HLC54)	[25]	*ura3*Δ*::imm434/ura3*Δ*::imm434 efg1*Δ*::hisG efg1*Δ*::hisG cph1*Δ*::hisG cph1*Δ*::URA3*
*tup1*Δ/Δ (BCa2-9)	[23]	*ura3*Δ*::imm434/ura3*Δ*::imm434 tup1*Δ*::hisG tup1*Δ*::URA3*
WT-GFP	This paper	*P_eno1_-EGFP_NAT^R^*
*edt1*Δ/Δ	This paper/[36]	*ura3*Δ*::imm434/ura3*Δ*::imm434 edt1*Δ*::hisG/edt1*Δ*::hisG::URA3::hisG*
edt1-GFP	This paper	*ura3*Δ*::imm434/ura3*Δ*::imm434 edt1*Δ*::hisG/edt1*Δ*::hisG::URA3::hisG P_eno1_-EGFP_NAT^R^*

All strains derived from the SC5314 clinical isolate.

### Alexa fluor labeling of live *C. albicans*

For Alexa fluor 647 labeling of *C. albicans*, cells pre-grown at 37°C overnight in YPD were washed three times in PBS and resuspended in PBS at a concentration of 1*10^9^/ml in 100 ul. Alexa fluor 647-succinimidyl ester (Molecular Probes) was dissolved and stored in DMSO at a concentration of 10 mg/ml. Just prior to labeling, 11 µl of 1 M NaCO_3_ pH 10 was added to the *Candida* and then 2 µl of Alexa fluor 647 was added and mixed briefly by vortexing. Cells were labeled for 1 hour at room temp, then washed five times with PBS, counted by hemacytometer, and used for infection or *in vitro* experiments. This resulted in no loss of viability, as assayed by broth dilution and plating to YPD.

### Infection and drug treatment of mice

For experiments lasting less than 24 hours, female BALB/c mice 7–10 weeks old were infected with 1*10^6^ *C. albicans* WT-GFP intravenously (i.v.) through the lateral tail vein. For experiments lasting more than 24 hours, the following doses were used to maximize infection burden without morbidity: 2.5*10^5^ (Day 3) or 1*10^5^ (Day 5 or 7). These doses gave kidney burdens within 1 order of magnitude at the time of euthanization: on days 1, 3, 5, and 7, kidney burdens averaged respectively 86, 22, 35, and 177 * 10^5^ colony forming units/g tissue. Therefore, the kidney burdens were comparable but the β-glucan unmasking was dramatically different. Animals were euthanized with CO_2_ 3 hours to 7 days post-infection, organs were dissected, and then weighed and homogenized in PBS with Dounce homogenizers. For colony forming unit assays, organ homogenates were serially diluted in 10-fold increments in PBS+penicillin/streptomycin and plated onto YPD plates in duplicate or triplicate. For drug treatment, 0.1 ml of a 0.6, 6, or 60 µg/ml solution of CF in PBS was injected per mouse i.p. just prior to tail vein infection, corresponding to doses of 3, 30, or 300 µg/kg. Alternatively, 0.1 ml of a 0.5 or 5 mg/ml solution of fluconazole (Sequoia Research Products, United Kingdom) in PBS was injected per mouse i.p. just prior to tail vein infection, corresponding to doses of 2.5 or 25 mg/kg. All animal experiments were conducted in accordance with NIH guidelines under IACUC Protocol # 0306-027-09.

### *Ex vivo* fluorescent staining (EVF)

Dounce-homogenized kidney tissue was washed three times with PBS, then mammalian cells were lysed with two washes of distilled water. For washes, cells were spun at 14,000×g for 30 seconds and pellets were vigorously resuspended with vortexing. Homogenates were washed again three times with PBS and then blocked with 2%BSA in PBS for 1 hour at room temp. Homogenates were then stained with anti-β-glucan antibody, specific for β1,3-glucan (Biosupplies, Inc., Australia) diluted 1∶200 in 2%BSA/PBS overnight at 4°C on a rotator and washed three times with PBS. Then homogenates were stained for 1 hour at room temp with goat anti-mouse Cy3 antibody (Jackson Immunoresearch) diluted 1∶100 into 2%BSA/PBS with or without Alexa647-labeled Dectin-CRD (diluted 1∶40 in 2%BSA/PBS). The carbohydrate recognition domain of mouse Dectin-1 (Dectin-CRD) was purified from *E. coli* and labeled as described previously [8]. After three washes, cells were visualized by fluorescence microscopy. Live cells were identified based on characteristic GFP fluorescence, with cytoplasmic fluorescence excluded only from vacuoles. Co-staining with propidium iodide shows that the small proportion of PI-positive cells (<1%) do not have characteristic GFP fluorescence, and vice versa (data not shown).

### Plasmid and *C. albicans* strain construction

Construction of the pENO1-yEGFP3-NAT plasmid was performed as follows. pENO1GFP3 [22] was digested with KpnI. The *ENO1* locus was amplified from SC5314 genomic DNA with forward primer (aattggccctgcagatgtcttacgccactaaaatccac) and reverse primer (aattggccggtaccccagcgtagatagcttcagaacct), which introduce KpnI and PstI sites, respectively. The *NAT1* gene was digested from pJK795 [34] with KpnI and PstI. A triple ligation of these two fragments with the KpnI-digested pENO1GFP3 resulted in pENO1-yEGFP3-NAT. EcoRV-digested plasmid was transformed into strain SC5314 by lithium acetate. Integrants were selected on 100 µg/ml nourseothricin (Werner Bioagents, Jena, Germany) and verified by PCR. This resulted in the WT-GFP strain, and transformation of the homozygous deletion strain *edt1::hisG/edt1::hisG-URA3-hisG* (a derivative of CAI4) yielded *edt1*-*GFP*.

### Fluorescence microscopy and Cellprofiler quantification

Samples were visualized with a Nikon TE2000-S microscope (Nikon, Tokyo, Japan) equipped with Spot RT camera (Diagnostic Instruments, Sterling Heights, MI, USA) and processed in Photoshop (Adobe Systems, Palo Alto, CA, USA). In Cellprofiler version 1.0.4884 (www.cellprofiler.org) 12-bit TIFF images of GFP fluorescence were used to manually define the edges of individual cell segments and 12-bit β-glucan fluorescence images without saturate pixels were used to quantify the average fluorescence intensity for the whole cell segment. Yeast form cells had a length-width ratio of <1.5 and characteristic shape and size. Average fluorescence values were analyzed using JMP 5 statistical software package. For fluorescence microscopy in supplementary figures, cells were imaged on an Olympus IX81 inverted microscope with 40× PlanApo objective using an Orca-ER cooled CCD camera (Hamamatsu Photonics) and using the IPLab v. 4.04 software package.

## Supporting Information

Figure S1 Several clinical isolates also display filament-specific exposure of β-glucan. Wildtype SC5314 (A–B) and clinical isolates CAN89 (C–D), CAN91 (E–F) and CAN92 (G–H) were grown overnight in YPD, washed, and diluted to 4*10^6^ cells/ml in fresh RPMI-PS with different concentrations of caspofungin or vehicle. After growth overnight at 30°C, cells grown in vehicle (B, D, F, and H) and at 1/2 MIC_50_ caspofungin (A, C, E, and G) were harvested and stained with anti-β-glucan antibody and Cy3-labeled secondary antibody. Cells were imaged by fluorescence microscopy and representative images were cropped and processed in Photoshop. Scale bar in (A) is 10 microns long and applies to all images.
(2.48 MB TIF)

Figure S2 Altering incubation times during staining procedure does not alter β-glucan exposure. WT-GFP *C. albicans* was pre-grown overnight in YPD, then diluted to 4*10^6^ cells/ml and grown overnight in RPMI-PS media at 30°C with either sub-inhibitory doses of caspofungin (Caspofungin; A–B, E–F, and I–J) or vehicle (Vehicle; C–D, G–H, and K–L), then washed with PBS. Cells were stained with normal length incubations (1 hour block, overnight primary antibody, 1 hour secondary antibody; A–D), short incubations (15 minutes block, 15 minutes primary antibody, 15 minutes secondary antibody; E–H), or extra-long incubations (overnight block, 24 hours primary antibody, 1 hour secondary antibody; I–L). Samples without primary antibody were used to identify non-specific staining. Cells were imaged by fluorescence microscopy and representative images were cropped and processed in Photoshop. Scale bar in (A) is 10 microns long and applies to all images.
(2.67 MB TIF)

Figure S3 Caspofungin exposes β-glucan but does not cause increased non-specific binding of mouse IgG antibody to *C. albicans*. WT-GFP *C. albicans* was grown overnight in YPD, washed, and diluted to 4*10^6^ cells/ml in fresh RPMI-PS with different concentrations of caspofungin or vehicle. After growth overnight at 30°C, cells grown in vehicle (E–H) and at 1/2 MIC_50_ caspofungin (A–D) were harvested and stained with anti-β-glucan antibody (Biosupplies, Inc; A and E), isotype control mouse IgG (Becton Dickinson; B and F), whole mouse IgG control (Santa Cruz Biotech; C and G) or no primary antibody (D and H) and Cy3-labeled goat anti-mouse IgG secondary antibody (Jackson Immunoresearch). Cells were imaged by fluorescence microscopy and representative images were cropped and processed in Photoshop. Scale bar in (A) is 10 microns long and applies to all images.
(4.56 MB TIF)

Figure S4 BSA blocking prevents non-specific antibody binding and doesn't cause extra β-glucan exposure. WT-GFP *C. albicans* was pre-grown overnight in YPD, then diluted to 4*10^6^ cells/ml and grown overnight in RPMI-PS media at 30°C with either sub-inhibitory doses of caspofungin (Caspofungin; A, C, and E) or vehicle (Vehicle; B, D, and F), then washed with PBS. (A–B) Cells were stained with a shortened protocol (30 minutes block, 30 minutes with primary, 15 minutes with secondary) using buffers including 2% BSA. (C–F) Washed cells were incubated for 24 hours at 4°C with 2% BSA to determine if BSA can expose β-glucan. Then, cells were washed and stained using the shortened protocol with (C–D) or without (E–F) BSA. Samples without primary antibody were used to identify non-specific staining. Cells were imaged by fluorescence microscopy and representative images were cropped and processed in Photoshop. Scale bar in (A) is 10 microns long and applies to all images.
(4.10 MB TIF)
