# Supplementary figures and images for: Dynamic, Morphotype-Specific Candida albicans β-Glucan Exposure during Infection and Drug Treatment
Source: PLoS Pathog. 2008 Dec 5;4(12):e1000227. doi: 10.1371/journal.ppat.1000227 (PMC2587227; doi:10.1371/journal.ppat.1000227)

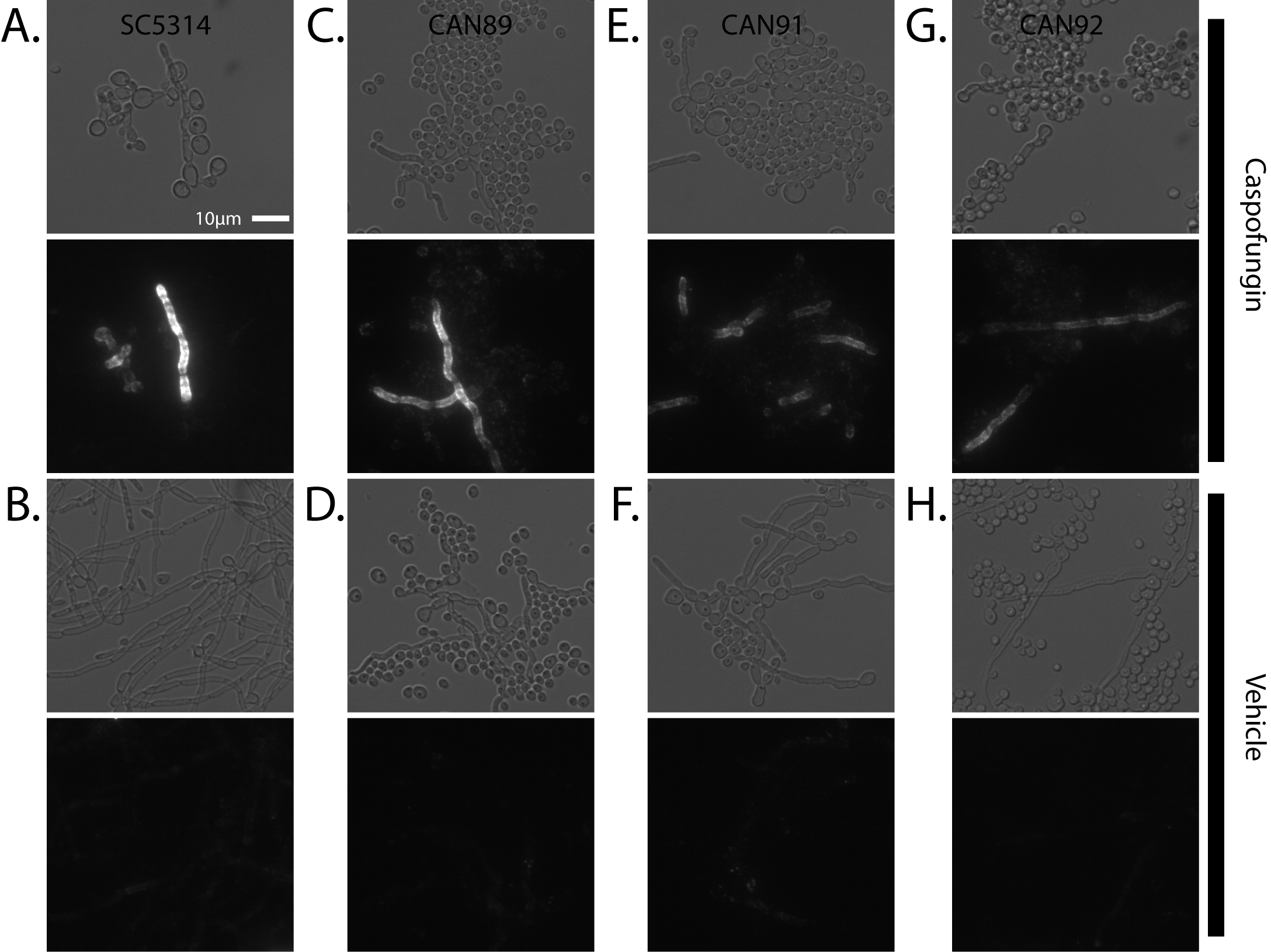

Supplement: Figure S1 — Several clinical isolates also display filament-specific exposure of β-glucan. Wildtype SC5314 (A–B) and clinical isolates CAN89 (C–D), CAN91 (E–F) and CAN92 (G–H) were grown overnight in YPD, washed, and diluted to 4*106 cells/ml in fresh RPMI-PS with different concentrations of caspofungin or vehicle. After growth overnight at 30°C, cells grown in vehicle (B, D, F, and H) and at 1/2 MIC50 caspofungin (A, C, E, and G) were harvested and stained with anti-β-glucan antibody and Cy3-labeled secondary antibody. Cells were imaged by fluorescence microscopy and representative images were cropped and processed in Photoshop. Scale bar in (A) is 10 microns long and applies to all images. (2.48 MB TIF) [file ppat.1000227.s001.tif]

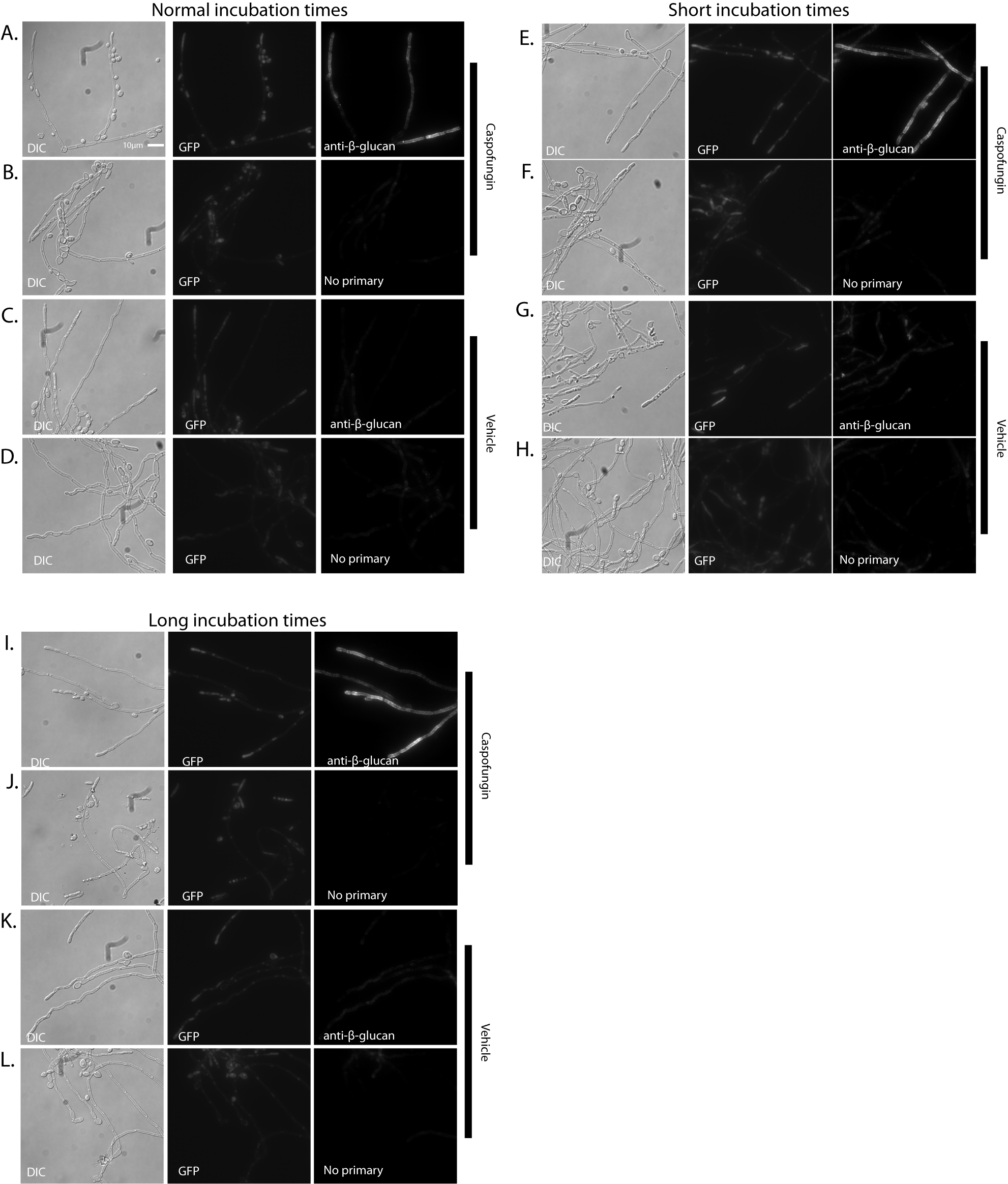

Supplement: Figure S2 — Altering incubation times during staining procedure does not alter β-glucan exposure. WT-GFP C. albicans was pre-grown overnight in YPD, then diluted to 4*106 cells/ml and grown overnight in RPMI-PS media at 30°C with either sub-inhibitory doses of caspofungin (Caspofungin; A–B, E–F, and I–J) or vehicle (Vehicle; C–D, G–H, and K–L), then washed with PBS. Cells were stained with normal length incubations (1 hour block, overnight primary antibody, 1 hour secondary antibody; A–D), short incubations (15 minutes block, 15 minutes primary antibody, 15 minutes secondary antibody; E–H), or extra-long incubations (overnight block, 24 hours primary antibody, 1 hour secondary antibody; I–L). Samples without primary antibody were used to identify non-specific staining. Cells were imaged by fluorescence microscopy and representative images were cropped and processed in Photoshop. Scale bar in (A) is 10 microns long and applies to all images. (2.67 MB TIF) [file ppat.1000227.s002.tif]

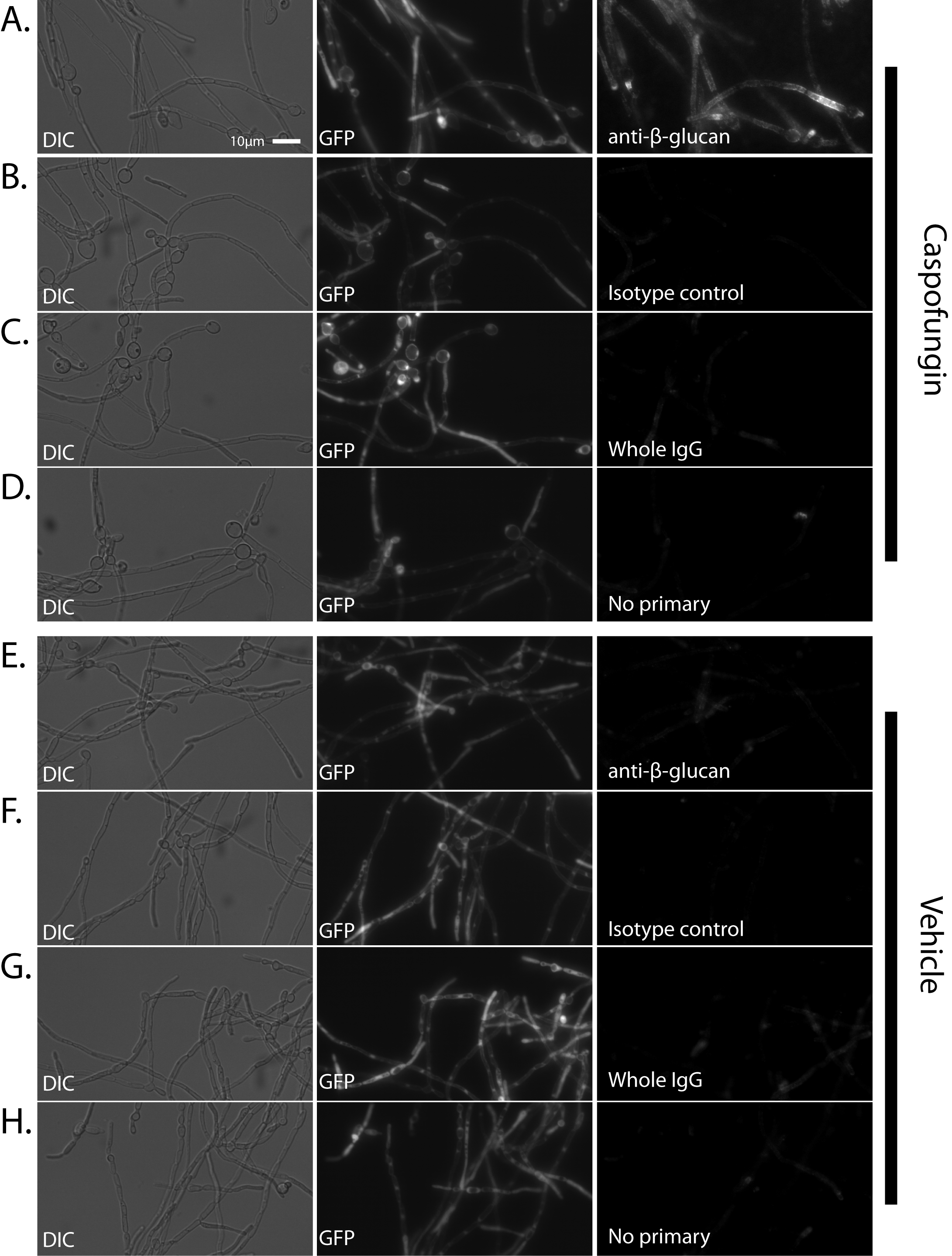

Supplement: Figure S3 — Caspofungin exposes β-glucan but does not cause increased non-specific binding of mouse IgG antibody to C. albicans. WT-GFP C. albicans was grown overnight in YPD, washed, and diluted to 4*106 cells/ml in fresh RPMI-PS with different concentrations of caspofungin or vehicle. After growth overnight at 30°C, cells grown in vehicle (E–H) and at 1/2 MIC50 caspofungin (A–D) were harvested and stained with anti-β-glucan antibody (Biosupplies, Inc; A and E), isotype control mouse IgG (Becton Dickinson; B and F), whole mouse IgG control (Santa Cruz Biotech; C and G) or no primary antibody (D and H) and Cy3-labeled goat anti-mouse IgG secondary antibody (Jackson Immunoresearch). Cells were imaged by fluorescence microscopy and representative images were cropped and processed in Photoshop. Scale bar in (A) is 10 microns long and applies to all images. (4.56 MB TIF) [file ppat.1000227.s003.tif]

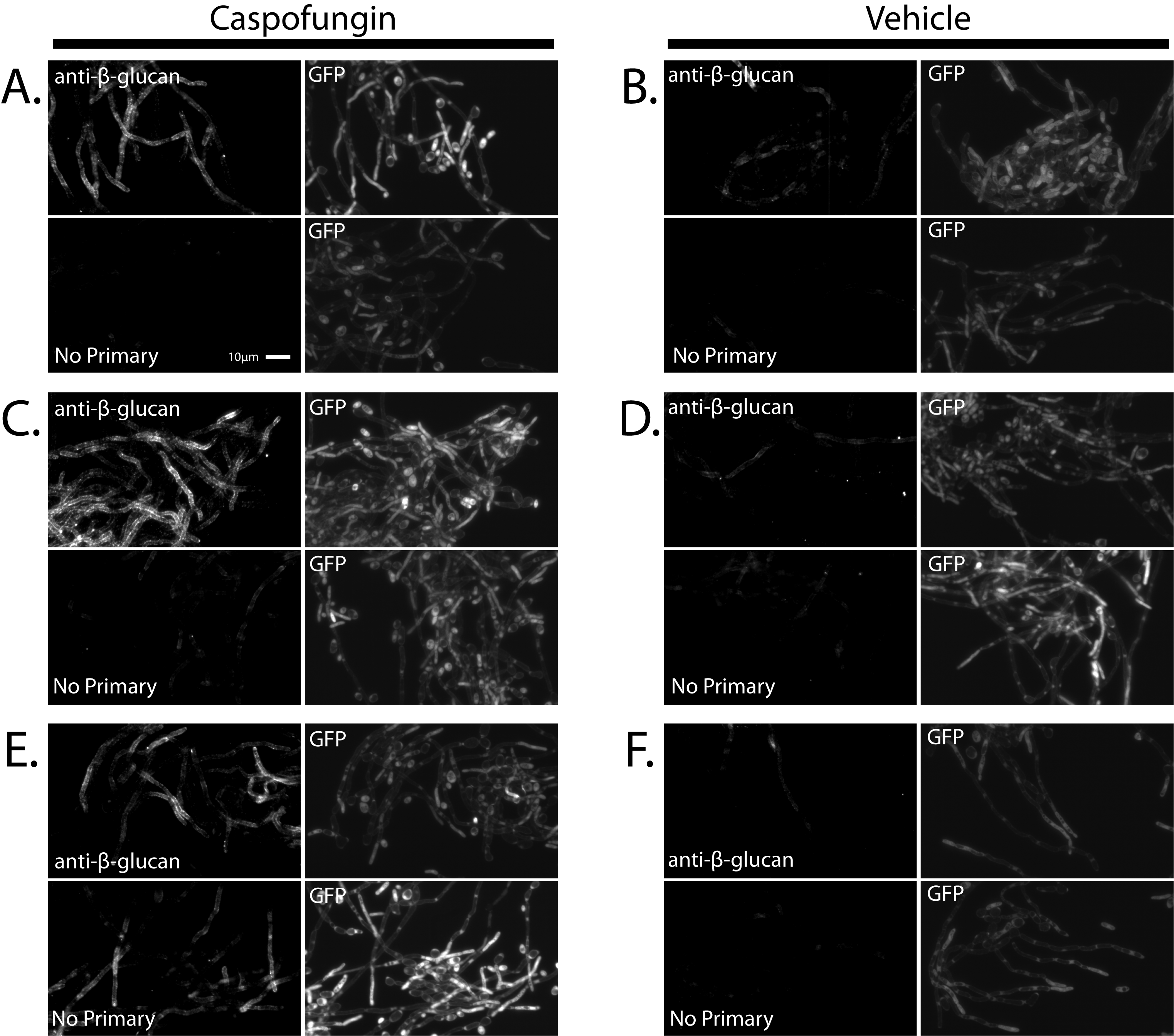

Supplement: Figure S4 — BSA blocking prevents non-specific antibody binding and doesn't cause extra β-glucan exposure. WT-GFP C. albicans was pre-grown overnight in YPD, then diluted to 4*106 cells/ml and grown overnight in RPMI-PS media at 30°C with either sub-inhibitory doses of caspofungin (Caspofungin; A, C, and E) or vehicle (Vehicle; B, D, and F), then washed with PBS. (A–B) Cells were stained with a shortened protocol (30 minutes block, 30 minutes with primary, 15 minutes with secondary) using buffers including 2% BSA. (C–F) Washed cells were incubated for 24 hours at 4°C with 2% BSA to determine if BSA can expose β-glucan. Then, cells were washed and stained using the shortened protocol with (C–D) or without (E–F) BSA. Samples without primary antibody were used to identify non-specific staining. Cells were imaged by fluorescence microscopy and representative images were cropped and processed in Photoshop. Scale bar in (A) is 10 microns long and applies to all images. (4.10 MB TIF) [file ppat.1000227.s004.tif]
